# What are pre‐service teachers like? Analysis of their psychosocial profile

**DOI:** 10.1111/sjop.12834

**Published:** 2022-06-02

**Authors:** Inmaculada García‐Martínez, José María Augusto‐Landa, Rocío Quijano‐López, Samuel P. León

**Affiliations:** ^1^ Department of Didactics and School Organization University of Granada Granada Spain; ^2^ Department of Psychology University of Jaén Jaén Spain; ^3^ Department of Education Science University of Jaén Jaén Spain; ^4^ Department of Pedagogy University of Jaén Jaén Spain

**Keywords:** Pre‐service teachers, emotional intelligence, resilience, personality, mental health

## Abstract

Modern society is becoming increasingly interested in people who are emotionally competent and who have the psychosocial skills required to be successful within the current social environment. However, no studies have been published on the assessment of the role of Emotional Intelligence (EI) on mental health if we assume the possible mediation of resilience and personality factors in the case of pre‐service teachers. Therefore, the aim of the study was to analyse the mediating role of resilience and personality factors in the relationship between EI and mental health of 1,022 pre‐service teachers enrolled in different educational degrees. The results found support the mediating role of resilience in the relationship between emotional intelligence and mental health; nevertheless, this was not the case when personality traits were analysed. Furthermore, it has been found that EI and resilience positively affect mental health of university students. Practical implications of this study are oriented towards an advance within the emerging trend of deepening the EI and resilience constructs among mental health care providers. Only if we understand the complex interactions between the constructs which determine people will it be possible to develop educational and health programmes responding to current needs.

## INTRODUCTION

The World Health Organization (2011) defines mental health as the state of well‐being in which a person performs individual abilities and is capable of coping with the common pressures of daily life, thus working productively and making a contribution to the community. In this positive regard, mental health is the basis for individual well‐being and effective community functioning.

There is little evidence on the demands and difficulties associated with university life from the perspective of students with mental health problems, although this perspective may be of great value in determining the best way to support these students (Inman, Moreira, Cunha, & Castro, 2020). University studies may be defined as a constantly changing period for students, involving the separation from family, high workloads, adaptation to new places and teachers, challenging assessments and high levels of academic stress (Freire, Ferradás, Núñez, Valle, & Vallejo, 2019). As a consequence, mental health of university students is an increasingly important concern worldwide. Authors such as Ingram and Luxon (2005) describe ways in which genetic, biological, psychological and cultural vulnerabilities interact with stressors, resulting in an increased probability for mental illness. The stressors to which university students are subjected are multiple and diverse. Among these, we can mention the need to achieve academic success, adaptation to new social networks and new environments, changes in the academic workload and family separation. This latter entails a great level of responsibility. Other factors to be mentioned here are the university accommodation itself or teacher‐related issues (de la Fuente *et al.,* 2020). Thus, stress presents severely negative effects on university students' mental health. Researchers have found that students with mental health problems obtain lower grades and experience higher drop‐out rates than their peers (García‐Escalera, Valiente, Sandín, Ehrenreich‐May, & Chorot, 2020).

As it has been stated by some authors, 12%–46% of university students are affected by mental health disorders in any of their years of study (Auerbach *et al.,* 2018). Most of the mental health disorders in life begin before university time (Kessler *et al.,* 2005) and once they arrive at university, these problems may be accelerated by the effect of academic stressors (Chacón‐Cuberos, Olmedo‐Moreno, Lara‐Sánchez, Zurita‐Ortega, & Castro‐Sánchez, 2019). Depression has been found to have increased exponentially and became the second leading cause of illness in 2020 (Santomauro *et al.,* 2021).). Mental disorders often shorten life (Hannerz, Borga, & Borritz, 2001). It is therefore essential to study those protective factors which may in turn change the way people cope with stressful events, thus helping to prevent the further development of mental disorders (Rutter, 2012). Among these protective factors, two of them will be specifically treated in this paper—resilience and emotional intelligence. Additionally, their direct and indirect contribution to the mental health of pre‐service teachers in the Faculties of Education in Andalusia (Spain) will be analysed.

### Resilience

Rutter (1993) took the term from physics and introduced it into psychology. According to this author, it may be defined as one self's ability to resist, to be strong and not to become distorted. Concerning human beings, resilience is the ability to overcome, to be strong, and to succeed against adversities. Thus, those who live high‐risk situations are more likely to be able to develop psychologically healthy and successfully (Rutter, 2012). There are several different definitions of this term. Perhaps one of the most accepted is the one proposed by Garmezy (1991), which defines it as the ability to recover and maintain an adaptive behaviour after the abandonment or the initial disability when a stressful event starts. According to Saavedra (2004) the resilient person is characterised by establishing constructive social relationships, having one’ s own positive sense of himself, measuring problems, displaying hope when faced with difficulties, having sense of initiative and setting possible goals to achieve.

Previous studies in the educational field have shown that resilience is positively associated with performance (Li, Li, & Li, 2019), with positive emotions (Chen & Padilla, 2019) and with the stressful situations that the students have to cope with, due to the changes that they experiment in the transition from secondary education to the university stressful period (Galindo‐Domínguez & Pegalajar, 2020). Other factors that affect resilience in the university population are related to age, maturity, work experiences, as well as other challenging experiences such as caregiving, which are associated with higher levels of resilience (Chung, Turnbull, & Chur‐Hansen, 2017). Furthermore, resilience is positioned as a key strength in understanding university success in relation to students' ability to adapt and grow from the challenges they have to overcome at university (Ayala & Manzano, 2018). Likewise, several studies have linked resilience to health, finding that highly resilient individuals develop confidence in themselves and their strengths and abilities. Additionally, they are able to manage stress and seem to be more enthusiastic and assertive. This undoubtedly results in a good mental health state (Brewer *et al.,* 2019; Frederick, Lobo, Chun, Wilfred, & Patrick, 2015). As Michael (2018) points out, resilient people look for supportive people, they laugh themselves and try to find good humour in their daily life situations, seek to have the moral courage to act appropriately, develop realistic plans to achieve their goals, etc… They also develop confidence in themselves and their strengths and abilities, manage stress (Pinar, Yildirim, & Sayin, 2018), they are enthusiastic and energetic, which results in good mental health.

### Emotional intelligence

The model proposed by Mayer and Salovey (1997) defines Emotional Intelligence as “the ability to perceive accurately, to value precisely and to express emotions properly; the ability to access and/or generate feelings that enable thinking; the ability to understand emotion and emotional knowledge and the ability to regulate emotions and to promote emotional and intellectual development” (Mayer & Salovey, 1997, p. 10). As pointed out by Brackett *et al*. (2013), the previously mentioned definition of EI is not directly related to emotions but instead to the integration of emotions with thoughts and behaviours, whereby one's behaviour tends to increase well‐being, quality of life and interpersonal functioning. Thus, a variety of cross‐sectional studies (Extremera & Fernández‐Berrocal, 2006; Fernández‐Abascal & Martín‐Díaz, 2015; Mikolajczak *et al.,* 2015) and meta‐analyses (Martins, Ramalho, & Morin, 2010; Sánchez‐Álvarez, Extremera, & Berrocal, 2016; Sarrionandia & Mikolajczak, 2020; Schutte, Malouff, Thorsteinsson, & Bhullar, 2007) have highlighted the important role that emotional intelligence skills play in individuals' health. It has been found that those people with high emotional skills report greater mental health and psychological well‐being. Other studies have highlighted the importance of EI for teachers in order to improve professional performance (Palomera, Briones, Gómez‐Linares, & Vera, 2017). Further research would benefit from the use of more diversified samples or themeasuring behavioural variables more objectively. Also, monitoring personality and systematically exploring the extent to which changes in EI (e.g., after training) lead in turn to changes in behaviour and/or biological parameters (Sarrionandia & Mikolajczak, 2020). Most of the above studies conclude that emotionally intelligent people are able to understand their own and others' emotions, cope with stress and develop strong and supportive relationships with others, which will result in good mental health.

### Personality traits

The Big Five personality traits (extraversion, neuroticism, conscientiousness, agreeableness and openness) are highly important within the field of psychology (Marengo, Sindermann, Elhai, & Montag, 2020). Thus, a set of meta‐analyses (Liu & Campbell, 2017; Oshio, Taku, Hirano, & Saeed, 2018) have shown that neuroticism is highly associated with the experience of negative emotions. Steel, Schmidt, and Shultz (2008) concluded in their meta‐analysis that the five personality traits can explain 39–63% of the variance in emotional well‐being. Likewise, high neuroticism, low consciousness, low agreeableness and low extraversion is a typical personality trait pattern associated with mental disorders. Studies that have related personality traits to psychological and social well‐being have found that psychological well‐being was negatively related to neuroticism and positively related to extraversion, agreeableness, and conscientiousness. The study by Joshanloo and Nosratabadi (2009) aimed to study the discriminative power of the Big 5 personality traits on mental health levels among Iranian university students. Their results found that individuals with different levels of mental health differ significantly in 4 of the 5 personality traits (extraversion, neuroticism, conscientiousness and agreeableness). A study by Lamers, Westerhof, Kovács, and Bohlmeijer (2012) found that extraversion and agreeableness were the only personality traits to be associated with mental health.

### Current study

WOS, Scopus and Google Scholar databases were consulted, where the keywords of the variables of the present study (personality, resilience, emotional intelligence and mental health) were entered to identify the background of the literature. No previous studies have been found concerning the possible mediation role of resilience and personality factors in that relationship and involving a sample of university students to assess the role of EI on mental health. Therefore, the aim of our study was to analyse the mediating role of resilience and personality factors in the relationship between EI and mental health of university students. In addition, the validity and internal consistency of the instruments used in this study will be analysed.

**Fig. 1:**
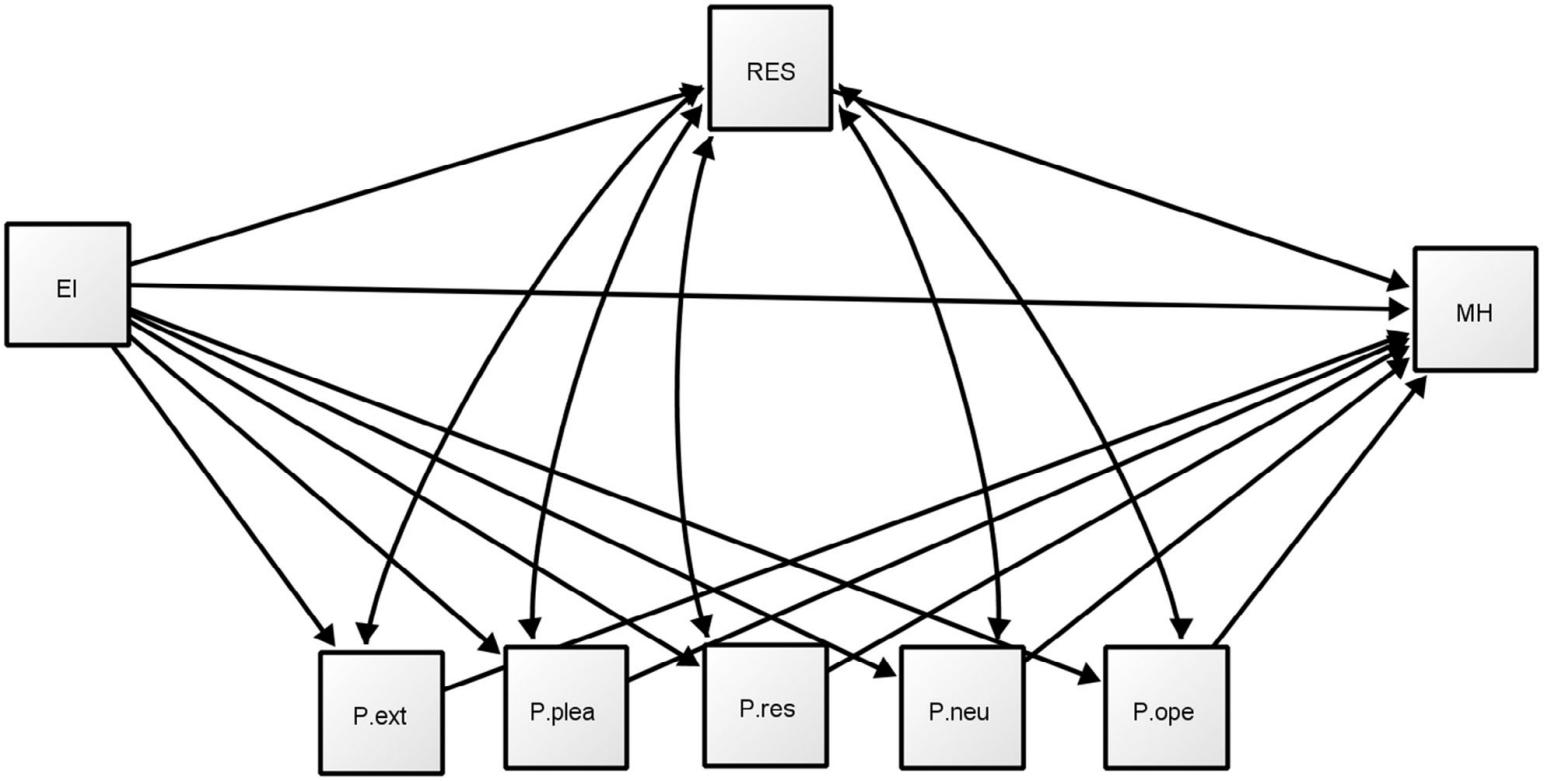
Proposed model of the determinants of relationship between mental health and Emotional Intelligence of university students.

The proposed model is shown schematically below (see Fig. 1).

The objectives of our study were the following:

Our first objective was to test for a direct relationship between EI and Mental Health. Our second objective was to test for a mediating relationship between EI, Resilience and Mental Health. Our third was to test whether personality traits mediate the relationship between EI, Resilience and Mental Health.

## METHOD

### Participants

The study was carried out by through non‐probability convenience sampling, in which students in the Bachelor's Degrees in Education from different universities in the south of Spain were invited to participate voluntarily. We calculated the minimal sample size at 95% confidence level, with a 5% confidence interval at 80% of statistical power (Hair, Black, Babin, & Anderson, 2010). In this regard, the estimated minimum sample size was 385.

The final sample resulting from the study involved 1,022 pre‐service teachers from different Faculties of Education in Andalusia. Concerning sex, it was found that 75.78% were women and 24.21% were men. In relation to age, participants were ranged from 17 to 50 years, with M 21.52 (SD 4.44). Regarding to their degree, 40.09% were enrolled in Primary Education, 26.76% in Early Childhood Education, 14.80% in Social Education, 6.37% were studying for a Master's degree in Teaching, 2.94% in Speech Therapy, and 9.04% were studying for other degrees. This range includes all individuals who were studying a degree whose frequency with respect to the total is less than 1%. Regarding the course, it was found that 57.05% were enrolled in the first year, 10% were in the second, 18. 72% in the third and 14.21% in the fourth. Finally, with regard to the region, it was found that 55.88% were studying in Jaén, followed by Granada (13.03%), Córdoba (10.09%), Cádiz (4.90%), Seville (4.41%), and Málaga (4.14%), and 7.55% belonged to other regions, whose frequency with respect to the total is less than 1%.

### Instruments

#### *Wong and Law emotional intelligence scale (WLEIS;* Wong & Law, 2002)

This scale is composed of 16 short sentences used to evaluate four dimensions: Self‐Emotion Appraisal (SEA), Other's Emotion Appraisal (OEA), Use of Emotion (UOE) and Regulation of Emotion (ROE). Participants in the study were asked to rate their agreement with the sentences on a five‐point Likert scale ranging from 1 (strongly disagree) to 5 (strongly agree). The Spanish version of Extremera, Rey, and Sánchez‐Álvarez (2019) was used. It has shown adequate validity and reliability in Spanish contexts (α = 0.91).

#### *Resilience scale (RS‐14,* Wagnild, 2009)

This instrument was designed to assess the individual's resilience through Equanimity, which refers to a balanced perspective on life and experiences. This could be seen as just sitting back and taking whatever comes, thus moderating extreme responses to adversities. This construction is often related to one's mood. RS‐14 scale validated by Sánchez‐Teruel and Robles‐Bello (2015) was used to determine resilience, which consists of 14 items, distributed in two dimensions: (a) Personal competence and (b) Self‐acceptance and life acceptance. The reliability analysis resulting from this scale was α = 0.93.

#### Big five Inventory‐44 (BFI‐44; Benet‐Martínez & John, 1998)

It is a self‐report test which measures the big five personality traits, i.e., extraversion, agreeableness, conscientiousness, neuroticism and openness. It is made up of 44 items with liker type responses ranging from 1 (total agreement) to 5 (total disagreement). This instrument was designed simultaneously in English and Spanish. The reliability analysis (Cronbach α) of this scale was for each of the corresponding factors of: extraversion (α = 0.69), agreeableness: (α = 0.75), conscientiousness (α = 0.74), neuroticism (α = 0.75) and openness (α = 0.77).

#### Mental health scale (MH‐5, Ware & Sherbourne, 1992)

This is a 5‐item reduced mental health scale based on the SF‐36 Health Questionnaire (Ware & Sherbourne, 1992), translated and adapted into Spanish by Alonso, Prieto, and Anto (1995). This instrument provides a health status profile which may be applied to the general population and also to patients. Specifically, it assesses individuals' depression and anxiety symptom levels over the past month. The range of responses ranges from 1 (“always”) to 6 (“never”). High scores are associated with better mental health. The reliability of the instrument was α = 0.77.

### Procedure

Before starting the questionnaire administration phase, the ethics committee of the University of Jaén (Spain) was asked to approve the implementation of this study and its approval was obtained, whose code is OCT.20/1.TES. Once ethical approval was obtained, the instrument was administered online, using the google form tool. In most cases, the authors of the manuscript attended classes to explain the purpose of the study and to guarantee the preservation of anonymity and ethics to potential participants. Once they agreed to participate voluntarily, they were provided with the link to the instrument. In those cases where it was not possible to attend classes, teachers were informed of the purpose of the research and provided with the researchers' contact details so that participants could contact them if they wished to do so.

### Data analysis

R programme were used for all the analyses performed within this study. In order to gain significance, the α value for all statistical tests was set to 0.05. Before factorial treatment, data was explored by data screening to analyse the assumptions required for factorial treatment and their distribution. A Confirmatory Factor Analysis (CFA) was performed to extract data resulting from each scale so as to verify the validity and internal consistency of those scales. CFA and SEM model analysis were performed using the r lavaan package (Rosseel, 2012). Because our data was not distributed in a normal multivariate way, the diagonally weighted minimum squares estimator (DWLS, Finney & DiStefano, 2013) was used. The Cronbach alpha and McDonald ω (Revelle, 2019) were used to study the reliability of the scales used. After factoring, original scores given by students in each questionnaire were then scaled by the standardised factor load obtained in CFA (Beaujean, 2014). Finally, the proposed theoretical model was analysed by SEM analysis.

## RESULTS

Table 1 shows the descriptive analysis results for each of the scales used in this research. Mardia's Multivariate Normality Test was performed to analyse multivariate normality. Results obtained showed that our data did not maintain a multivariate normal distribution (*Z*_*Kurtosis*_ = 81.62, *p* < 0.01).

**Table 1:** Descriptives for items

Items	N	Missing	*M*	*Me*	*SD*	Min	Max	Skewness	Kurtosis
re1	1,020	0	5.54	6.00	1.03	2	7	−0.52	0.08
re2	1,020	0	6.21	7.00	1.02	1	7	−1.59	3.17
re3	1,020	0	4.61	5.00	1.49	1	7	−0.20	−0.57
re4	1,020	0	4.62	5.00	1.51	1	7	−0.53	−0.26
re5	1,020	0	4.73	5.00	1.38	1	7	−0.35	−0.24
re6	1,020	0	4.80	5.00	1.42	1	7	−0.39	−0.28
re7	1,020	0	4.49	5.00	1.63	1	7	−0.22	−0.78
re8	1,020	0	5.29	5.00	1.33	1	7	−0.77	0.37
re9	1,020	0	5.82	6.00	1.10	1	7	−1.07	1.37
re10	1,020	0	5.89	6.00	1.16	1	7	−1.01	0.74
re11	1,020	0	4.77	5.00	1.59	1	7	−0.49	−0.48
re12	1,020	0	6.33	7.00	0.95	1	7	−1.80	4.05
re13	1,020	0	5.80	6.00	1.39	1	7	−1.32	1.55
re14	1,020	0	5.39	6.00	1.16	1	7	−0.77	0.65
ei1	1,020	0	2.85	3.00	0.81	0	4	−0.53	0.43
ei2	1,020	0	2.78	3.00	0.84	0	4	−0.56	0.38
ei3	1,020	0	2.76	3.00	0.87	0	4	−0.34	−0.29
ei4	1,020	0	3.03	3.00	0.92	0	4	−0.64	−0.32
ei5	1,020	0	2.98	3.00	0.78	0	4	−0.51	0.23
ei6	1,020	0	3.16	3.00	0.82	0	4	−0.83	0.53
ei7	1,020	0	3.28	3.00	0.83	0	4	−1.00	0.45
ei8	1,020	0	3.16	3.00	0.73	0	4	−0.60	0.21
ei9	1,020	0	2.95	3.00	0.91	0	4	−0.57	−0.26
ei10	1,020	0	2.56	3.00	0.98	0	4	−0.34	−0.34
ei11	1,020	0	2.59	3.00	1.07	0	4	−0.44	−0.46
ei12	1,020	0	2.76	3.00	1.03	0	4	−0.58	−0.25
ei13	1,020	0	2.50	3.00	1.02	0	4	−0.36	−0.33
ei14	1,020	0	2.44	3.00	0.96	0	4	−0.39	−0.17
ei15	1,020	0	2.10	2.00	1.10	0	4	−0.10	−0.66
ei16	1,020	0	2.40	2.00	0.92	0	4	−0.24	−0.19
pe1	1,020	0	3.57	4.00	0.97	1	5	−0.40	−0.06
pe2	1,020	0	2.48	2.00	1.04	1	5	0.35	−0.55
pe3	1,020	0	3.59	4.00	0.99	1	5	−0.35	−0.36
pe4	1,020	0	2.17	2.00	1.13	1	5	0.73	−0.36
pe5	1,020	0	3.54	4.00	0.96	1	5	−0.24	−0.43
pe6	1,020	0	3.09	3.00	1.17	1	5	−0.05	−0.78
pe7	1,020	0	4.37	4.00	0.71	1	5	−0.93	0.58
pe8	1,020	0	3.17	3.00	1.14	1	5	−0.11	−0.75
pe9	1,020	0	2.99	3.00	1.07	1	5	−0.03	−0.49
pe10	1,020	0	3.70	4.00	0.90	1	5	−0.20	−0.47
pe11	1,020	0	3.67	4.00	0.95	1	5	−0.41	−0.11
pe12	1,020	0	2.95	3.00	1.10	1	5	−0.01	−0.56
pe13	1,020	0	1.83	2.00	0.98	1	5	1.06	0.44
pe14	1,020	0	4.12	4.00	0.84	1	5	−0.70	0.07
pe15	1,020	0	2.76	3.00	1.08	1	5	0.20	−0.63
pe16	1,020	0	2.82	3.00	1.24	1	5	0.12	−0.94
pe17	1,020	0	3.46	3.00	1.10	1	5	−0.24	−0.70
pe18	1,020	0	2.58	3.00	1.27	1	5	0.29	−1.00
pe19	1,020	0	2.96	3.00	1.07	1	5	−0.03	−0.58
pe20	1,020	0	3.62	4.00	1.00	1	5	−0.33	−0.54
pe21	1,020	0	3.86	4.00	0.95	1	5	−0.49	−0.35
pe22	1,020	0	1.75	1.00	0.95	1	5	1.21	0.83
pe23	1,020	0	3.10	3.00	1.10	1	5	−0.07	−0.59
pe24	1,020	0	3.49	4.00	0.99	1	5	−0.39	−0.29
pe25	1,020	0	2.36	2.00	1.16	1	5	0.52	−0.62
pe26	1,020	0	4.02	4.00	0.93	1	5	−0.71	0.03
pe27	1,020	0	3.11	3.00	1.21	1	5	−0.11	−0.88
pe28	1,020	0	3.23	3.00	1.18	1	5	−0.22	−0.78
pe29	1,020	0	3.76	4.00	0.80	1	5	−0.33	0.07
pe30	1,020	0	2.78	3.00	1.11	1	5	0.12	−0.69
pe31	1,020	0	3.37	3.00	0.89	1	5	−0.02	−0.18
pe32	1,020	0	3.42	3.00	0.93	1	5	−0.18	−0.22
pe33	1,020	0	2.63	3.00	1.17	1	5	0.24	−0.82
pe34	1,020	0	3.39	3.00	0.98	1	5	−0.37	−0.30
pe35	1,020	0	3.37	3.00	0.98	1	5	−0.21	−0.38
pe36	1,020	0	3.66	4.00	0.94	1	5	−0.18	−0.66
pe37	1,020	0	4.17	4.00	0.81	1	5	−0.81	0.54
pe38	1,020	0	3.24	3.00	1.12	1	5	−0.12	−0.73
pe39	1,020	0	3.04	3.00	1.18	1	5	0.02	−0.79
pe40	1,020	0	3.34	3.00	1.07	1	5	−0.23	−0.50
pe41	1,020	0	3.96	4.00	0.91	1	5	−0.71	0.25
pe42	1,020	0	3.33	3.00	1.06	1	5	−0.13	−0.57
pe43	1,020	0	3.70	4.00	1.04	1	5	−0.51	−0.32
pe44	1,020	0	2.56	3.00	1.11	1	5	0.21	−0.67
mh1	1,020	0	3.08	3.00	1.08	1	5	−0.11	−0.54
mh2	1,020	0	3.46	4.00	1.19	1	5	−0.29	−0.88
mh3	1,020	0	2.80	3.00	1.01	1	5	0.12	−0.53
mh4	1,020	0	3.31	3.00	1.17	1	5	−0.24	−0.80
mh5	1,020	0	2.59	2.00	1.21	1	5	0.38	−0.80

*Notes*: Re = resilience; Ei = Emotional Intelligence; Pe = Personality; Mh = Mental Health.

A data screening was performed before factorial treatment to explore their distribution and to analyse assumptions. The correlation of the variables to analyse additivity showed that our data did not show multicollinearity (*r* > 0.90), nor singularity (*r* > 0.95). To analyse linearity, homogeneity and homoscedasticity, a linear regression with obtained data and randomly created data sets was performed. Subsequently, the residues of this regression were explored. It was stated that any anomaly in the distribution of the residues would be due to the distribution of our data, given the fact that the other variable was random (Kline, 2015). The distribution of the residues did not show any anomaly, being this mostly distributed between −2 and + 2.

### Analysis of the subscales

For the purpose of analysing the validity and internal consistency of the scales used in the study, a CFA was performed with each of the data sets obtained with each of the scales. The results of each CFA are presented below.

#### Resilience scale (RS‐14)

Standardised factor loads for this scale varied between .377 (SE 0.016) and .728 (SE 0.019), for more details see Table 2. The CFA for *RS‐14* scale showed an excellent fit (Hair *et al.,* 2010), *χ* ^*2*^ (77) = 279.935, *p* < .001, with CFI = 0.972, TLI = 0.967, SRMR = 0.063, RMSEA = 0.051 (RMSEA 90% CI [0.045, 0.057]). The reliability of this scale was Cronbach α = 0.867 and McDonald ω = 0.868.

**Table 2:** Factor loading

Scale	Latent Factor	Indicator	Estimate	*SE*	*Z*	*p*	Stand. Estimate
RES	res	re1	0.593	0.018	32.326	< 0.001	0.593
		re2	0.492	0.020	24.064	< 0.001	0.492
		re3	0.377	0.016	22.862	< 0.001	0.377
		re4	0.695	0.019	37.464	< 0.001	0.695
		re5	0.719	0.019	37.901	< 0.001	0.719
		re6	0.728	0.019	38.972	< 0.001	0.728
		re7	0.493	0.017	29.496	< 0.001	0.493
		re8	0.499	0.018	27.286	< 0.001	0.499
		re9	0.447	0.018	25.536	< 0.001	0.447
		re10	0.493	0.019	26.391	< 0.001	0.493
		re11	0.701	0.018	37.958	< 0.001	0.701
		re12	0.371	0.018	20.649	< 0.001	0.371
		re13	0.527	0.019	28.026	< 0.001	0.527
EI	se	ei1	0.702	0.023	30.157	< 0.001	0.702
		ei2	0.800	0.025	32.181	< 0.001	0.800
		ei3	0.761	0.023	32.490	< 0.001	0.761
		ei4	0.544	0.020	26.560	< 0.001	0.544
	oth	ei5	0.666	0.034	19.565	< 0.001	0.666
		ei6	0.778	0.037	20.754	< 0.001	0.778
		ei7	0.389	0.027	14.517	< 0.001	0.389
		ei8	0.749	0.036	20.527	< 0.001	0.749
	use	ei9	0.486	0.021	23.098	< 0.001	0.486
		ei10	0.725	0.024	30.758	< 0.001	0.725
		ei11	0.845	0.026	33.019	< 0.001	0.845
		ei12	0.850	0.026	33.152	< 0.001	0.850
	reg	ei13	0.722	0.022	33.002	< 0.001	0.722
		ei14	0.847	0.023	36.468	< 0.001	0.847
		ei15	0.578	0.020	28.825	< 0.001	0.578
BF	ext	pe1	0.486	0.017	27.797	< 0.001	0.486
		pe11	0.668	0.019	35.129	< 0.001	0.668
		pe27	0.417	0.017	24.895	< 0.001	0.417
		pe32	0.665	0.019	35.129	< 0.001	0.665
		pe40	0.493	0.017	28.241	< 0.001	0.493
		pe43	0.626	0.018	33.851	< 0.001	0.626
	agr	pe7	0.459	0.019	24.824	< 0.001	0.459
		pe24	0.426	0.018	23.388	< 0.001	0.426
		pe33	0.383	0.018	21.776	< 0.001	0.383
		pe37	0.554	0.020	27.592	< 0.001	0.554
		pe41	0.609	0.021	29.384	< 0.001	0.609
	con	pe14	0.631	0.022	28.959	< 0.001	0.631
		pe21	0.697	0.022	31.016	< 0.001	0.697
		pe25	0.411	0.019	21.536	< 0.001	0.411
		pe29	0.603	0.021	28.741	< 0.001	0.603
		pe34	0.415	0.019	21.316	< 0.001	0.415
	neu	pe4	0.566	0.021	26.471	< 0.001	0.566
		pe9	0.575	0.022	25.812	< 0.001	0.575
		pe15	0.613	0.022	27.704	< 0.001	0.613
		pe19	0.582	0.022	25.877	< 0.001	0.582
		pe30	0.522	0.021	24.620	< 0.001	0.522
		pe35	0.532	0.022	24.188	< 0.001	0.532
		pe38	0.550	0.022	25.390	< 0.001	0.550
	ope	pe5	0.747	0.019	39.422	< 0.001	0.747
		pe10	0.444	0.017	25.739	< 0.001	0.444
		pe20	0.764	0.019	39.223	< 0.001	0.764
		pe23	0.633	0.018	34.490	< 0.001	0.633
		pe31	0.543	0.018	29.994	< 0.001	0.543
		pe36	0.628	0.018	34.489	< 0.001	0.628
MH	mh	mh1	0.501	0.025	19.950	< 0.001	0.501
		mh2	0.801	0.030	26.995	< 0.001	0.801
		mh3	0.584	0.026	22.737	< 0.001	0.584
		mh4	0.790	0.030	26.628	< 0.001	0.790
		mh5	0.588	0.025	23.362	< 0.001	0.588

*Notes*. RES: Resilience; EI: Emotional intelligence; BF: Big Five; MH: Mental Health; re: resilience; se: Self‐Emotion Appraisal, oth: Other's Emotion Appraisal, use: Use of Emotion; reg: Regulation of Emotion; ext.: extraversión; agr: agreeableness, con: conscientiousness; neu: neuroticism; ope: openness.

#### Wong Law emotional intelligence scale (WLEIS‐S)

Standardised factor loads for this scale varied between 0.389 (SE 0.027) and 0.890 (SE 0.024), for more details see Table 2. The CFA for *WLEIS‐S* scale showed an excellent fit (Hair *et al.,* 2010), *χ* ^*2*^ (98) = 183.180, *p* < 0.001, with CFI = 0.989, TLI = 0.987, SRMR = 0.041, RMSEA = 0.029 (RMSEA 90% CI [0.023, 0.036]). The reliability of this scale was Cronbach α = 0.834 and McDonald ω = 0.894.

#### Personality scale (Big5)

Analysis of the original scale produced an unacceptable fit (Hair *et al.,* 2010), *χ* ^*2*^ (892) = 7238.382, *p* < 0.001, with CFI = 0.746, TLI = 0.892, SRMR = 0.090, RMSEA = 0.084 (RMSEA 90% CI [0.082, 0.085]). Since this scale is one of the most widely used scales in the study of personality (Barrick & Mount, 1991), we decided to eliminate the items with standardised factor loads below 0.3. With the resulting model, the analysis was performed one more time. Standardised factor loads for this scale varied between 0.383 (SE 0.018) and 0.764 (SE 0.019), for more details see Table 2. The CFA for *Big5* scale showed a good fit (Hair *et al.,* 2010), *χ* ^*2*^ (367) = 1759.512, *p* < 0.001, with CFI = 0.908, TLI = 0.898, SRMR = 0.067, RMSEA = 0.061 (RMSEA 90% CI [0.058, 0.064]). The reliability of this scale was Cronbach's α = 0.763 and McDonald's ω = 0.823.

#### Mental health scale (MH‐5)

Standardised factor loads for this scale varied between 0.501 (SE 0.025) and .801 (SE 0.030), for more details see Table 2. The CFA for *MH‐5* scale showed an excellent fit (Hair *et al.,* 2010), *χ* ^*2*^ (5) = 55.384, *p* < 0.001, with CFI = 0.972, TLI = 0.944, SRMR = 0.065, RMSEA = 0.099 (RMSEA 90% CI [0.077, 0.124]). The reliability of this scale was Cronbach α = 0.787 and McDonald ω = 0.792.

### Structural equation model

The proposed structural model is presented in Fig. 1. In this figure, the squares represent the values of the scaled variables obtained from each of the scales. The arrows in one direction indicate regression relationships while the arrows in two directions indicate correlation relationships. The results of SEM analysis for the proposed model showed an excellent fit of the model (Hair *et al.,* 2010), *χ* ^*2*^ (10) = 168,993, *p* < 0.001, with IFC = 0.935, TLI = 0.816, SRMR = 0.091, RMSEA = 0.125 (RMSEA 90% CI [0.109, 0.142]).

**Fig. 2:**
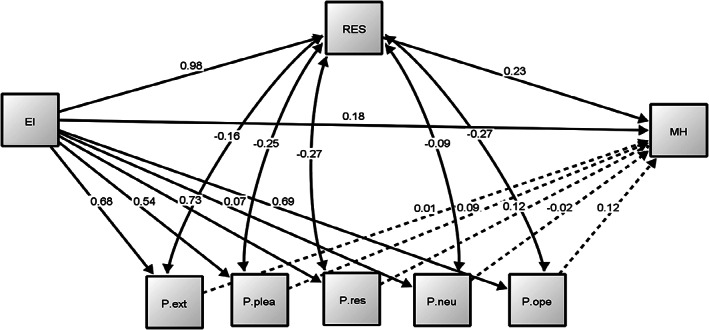
SEM Model.

Table 3 presents the complete results of the SEM analysis of the structural model presented in Fig. 2.

**Table 3:** Result from hypothesised model

Latent Variables	Estimate	*SE*	*Z*	*p*	Stand. Estimate
EI ~					
RES	1.296	0.109	11.908	< 0.001	0.980
P.ext	0.905	0.048	19.033	< 0.001	0.684
P.agre	0.717	0.040	17.818	< 0.001	0.542
P.con	0.960	0.050	19.129	<0 .001	0.725
P.neu	0.094	0.029	3.244	0.001	0.071
P.ope	0.917	0.047	19.33	<0 .001	0.693
EI~					
MH	0.183	0.035	5.289	< 0.001	0.183
MH ~					
RES	0.231	0.053	4.393	< 0.001	0.230
P.ext	0.008	0.097	0.083	0.934	0.008
P.agr	0.087	0.122	0.713	0.476	0.087
P.con	0.122	0.170	0.718	0.473	0.122
P.neu	−0.023	0.056	−0.417	0.676	−0.023
P.ope	0.116	0.160	0.723	0.470	0.116
RES ~ ~					
P.ext	−0.160	0.066	−2.418	0.016	−0.160
P.agr	−0.252	0.057	−4.395	<0 .001	−0.252
P.con	−0.267	0.068	−3.900	< 0.001	−0.267
P.neu	−0.088	0.038	−2.287	0.022	−0.088
P.ope	−0.268	0.065	−4.115	<0 .001	−0.268

*Note:* EI = Emotional intelligence; RES = Resilience; P.ext. = extraversion; P.agr = agreeableness; P.con = conscientiousness; P.neu = neuroticism; P.ope = openness; MH = Mental Health. ~ indicates regression relationship; ~ ~ indicates correlation relationship.

The regression relationships are shown in the upper part and the correlation relationships are shown in the lower part. The significant relationships are represented in Fig. 1 with black arrows, while the non‐significant relationships seem to be represented in the figure with dotted arrows. As can be seen, Emotional Intelligence (EI) and Resilience (RES) have a very high relationship (*β* = 0.980), RES and mental health (MH) also showed a significant positive relationship (*β* = 0.230). The direct relationship between EI and MH was shown to be significant (*β* = 0.183). The results shown so far indicate that EI is able to predict part of the variance of students' MH scores directly. Additionally, the results shown by our explanatory model indicate that IE can also predict part of the variance of the MH scores through the mediation of the RES.

In the case of the personality traits, all showed significant regression relationships with EI (highest P.res, *β* = 0.725), whereas no personality traits showed any significant relationship with mental health (MH). These results show the close relationship between EI and the personality factors measured by the Big5 scale (note that, as expected, in the case of Neuroticism the relationship, despite being significant, shows a standardised weight close to 0). But unlike the mediating role we have seen RES play in explaining MH scores, none of the personality factors helped predict MH. Finally, all personality traits showed significant and negative correlations with RES.

## DISCUSSION

The main objective of this study was to analyse the relationships between EI, Resilience, and personality traits on mental health in pre‐service teachers from Education degrees. Thus, we found significant and highly positive relationships between EI and Resilience. This finding is consistent with other work (Magnano, Craparo, & Paolillo, 2016; Sarrionandia, Ramos‐Díaz, & Fernández‐Lasarte, 2018). These findings reveal that individuals who are able to understand and manage their own and others' emotions appear to have a predictable impact on their high resilience. It was also found that Resilience had a positive impact on students' mental health. These results are consistent with other studies which show an important correlation with psychological well‐being (Harms, Brady, Wood, & Silard, 2018). Likewise, Connor and Davidson (2003) report that resilient people may keep their psychological health by cushioning the negative effects produced in challenging situations. As stated by Ungar and Theron (2020), resilience may be best understood as the process of multiple biological, psychological, social and ecological systems interacting to assist people to recover, maintain or improve their mental well‐being when faced with challenges caused by one or more risk factors. Moreover, the results have shown evidence of an indirect role for EI over resilience in improving mental health. In this regard, the studies developed by Galindo‐Domínguez and Pegalajar (2020), Cejudo, López‐Delgado, and Rubio (2016) and Magnano *et al*. (2016) have shown the role of EI on resilience, showing that those individuals with higher EI exhibit greater resilience. Personality traits did not directly explain the mental health variable. According to Lamers *et al*. (2012), studies which link personality traits and mental health show relationships between personality components and positive mental health. However, these are studies which directly compare the single relationship of personality with the components of mental health. As the authors state, more studies are required to analyse the role of personality components along with other variables included within this study. Although the variables of EI and resilience have been used in other studies, there have been no other studies so far which have highlighted the relationships of these constructs with mental health in pre‐service teachers. The most important contribution of this study, which analysed the relationship between EI and health, was therefore to verify that resilience was established as a mediating factor in this relationship among pre‐service teachers enrolled in Education degrees in Andalusia (Spain). These results confirm that resilience plays a key role in the students' mental health.

We will now point out some of the limitations of this study: the design of this study is cross‐sectional, which does not allow the establishment of any causal effect between the studied factors. Further studies will have to consider mediational models using longitudinal design to obtain a better understanding of the associations between these variables. Another limitation of this study is given by the distribution of the sample in terms of gender. There was majority of female individuals when compared to male. This is partially due to the fact that in general, there are more female students than male students in all Spanish Education degrees. Further studies should have other degrees in which there are more male subjects in order to equal percentages by gender. Regarding the tests used, future studies should use, as far as possible, performance tests, in order to obtain information on possible differences between variables and thus reducing the influence of subjectivity. From data obtained in this study it can be concluded that EI and resilience enhance mental health in pre‐service teachers. From these results, important practical implications for this group can be drawn. Due to the growing interest in the constructs of EI and resilience among mental health care providers, there is a need to recognise the complex interactions between the systems specifically designed to predict which people will do well and use this information to promote mental health interventions. In conclusion, the implementation of EI and resilience intervention programmes are essential in order to test their impact on individuals' mental health.

## DECLARATIONS

• Conflicts of interest/Competing interests: Not applicable.
• Funding: Funding for open access charge: Universidad de Granada/CBUA. This research is supported by Research Plan of the University of Granada. Title of the project “Pre‐service Teacher professionalisation in social emotional skills in the post‐pandemic period” (Grant ID: PPJIA2021‐28). IP: Inmaculada García‐Martínez.
• Availability of data and material: Data are available on justified request to the corresponding author.
• Ethics approval: This research has been approved by the Ethics Committee of the University of Jaén. Code: OCT.20/1.TES.
• Consent to participate: The authors gave their consent to participate voluntarily in the research.
• Acknowledgements: The research is included within the Ibero‐American Network for the Development of Professional Teaching Identity.
• Funding for open access charge: University of Granada / CBUA.

## Data Availability

Data sharing is not applicable to this article as no new data were created or analyzed in this study.
